# Joint effusion, anteroposterior stability, muscle strength and degree of patellofemoral osteoarthritis significantly impact outcome following revision ACL reconstruction

**DOI:** 10.1186/s40634-021-00370-x

**Published:** 2021-08-26

**Authors:** Kathleen Andrä, Enes Kayaalp, Robert Prill, Lars Irlenbusch, Eckehard Liesaus, Tilo Trommer, Peter Ullmann, Roland Becker

**Affiliations:** 1 Center of Orthopaedics and Traumatology, University of Brandenburg, Medical School “Theodor Fontane”, Hochstrasse 29, 14770 Brandenburg an der Havel, Germany; 2 Sports Clinic Erfurt, Erfurt, Germany; 3 Department of Orthopedics and Traumatology, Istanbul Taksim Training and Research Hospital, Siraselviler Cad. 34433 Beyoglu, Istanbul, Turkey

**Keywords:** Anterior cruciate ligament reconstruction, Revision, IKDC, Tegner activity scale, Effusion, Quadriceps, Instability

## Abstract

**Abstract:**

Purpose: Effusion, impaired muscle function and knee instability are considered as some of the most important factors effecting outcome following anterior cruciate ligament reconstruction (ACL-R) but the impact on revision ACL-R remains unclear. It was hypothesized that these factors will significantly worsen clinical outcome following revision ACL-R.

**Methods:**

Seventy knees (13 female and 57 male) were followed retrospectively after revision ACL-R at a mean follow-up of 47.8 ± 20.7 months. Clinical examination was based on the International Knee Documentation Evaluation Form-2000 (IKDC), Tegner activity scale. Instrumented measurement of anterior tibial translation was performed using the Rolimeter® (DJO Global, Freiburg, Germany). Bilateral circumference of the thigh was measured 10 and 20 cm proximal to the medial joint space. Cartilage was assessed according to Outerbridge classification during both primary and revision ACL-R.

**Results:**

Tegner activity scale decreased significantly from 7.8 ± 1.4 points at primary ACL-R to 7 ± 1.8 points at revision ACL-R, and 5.8 ± 1.7 points at the time of follow up (*p* < 0.001). Joint effusion (*r* = − 0.47, *p* < 0.01) and side to side differences in single leg hop test (*r* = − 0.48, *p* < 0.1) significantly correlated with inferior outcome. Cartilage lesions were found in 67% of the patients at the time of revision ACL-R compared to 38% at the time of primary ACL-R. According to the IKDC classification A was graded in three patients (4.3%), B in 35 (50%), C in 29 (41.4%) and D in three (4.3%). Joint effusion was measured in 35% of patients at the time of follow-up. Degeneration at the patellofemoral compartment of > grad 2 was responsible for IKDC grade C and D (*p* = 0.035). Instrumented anteroposterior site-to-site difference of ≥3 mm showed significant impact on clinical outcome (*p* < 0.019).

**Conclusion:**

The study has shown that chronic effusion, quadriceps dysfunction, cartilage lesions especially at the patellofemoral compartment and side to side difference in anteroposterior stability significantly influences patient outcome after revision ACL-R. These factors require special attention when predicting patient’s outcome.

**Level of evidence:**

Level-IV, case-controlled study.

## Introduction

Consensus criteria defined for successful outcome following anterior cruciate ligament reconstruction (ACL-R) including effusion, laxity, muscle strength, functional performance and patients reported outcome [26]. However, it remains unclear whether the criteria developed for outcome assessment following primary ACL-R may be applicable for revision ACL-R.

Patients and surgery related factors were shown to influence the outcome following primary and revision ACL-R. For example, the incidence of osteoarthritis (OA) is increased after non-anatomical ACL-R in comparison with anatomical ACL-R (23.2% vs 43.9%) after 10 years of follow up [31]. The type of graft does not seem to have an impact on knee stability following primary or revision ACL-R comparing the use of hamstring tendons [semitendinosus/gracilis (STG)], bone-patellar tendon-bone (BTB), or Achilles tendon allograft [1]. However, a meta-analysis of the literature showed better outcome when using autograft compared to allograft [13].

Numerous factors influence clinical and functional outcome following primary and revision ACL-R. While some authors reported no difference in muscle function and clinical outcome between primary and revision ACL-R others showed significant difference in muscle strength, patient related outcome measures and in frequency of return to sport [6, 23]. Lefevre et al. found worse outcome following revision ACL-R based on clinical and functional assessment but a comparable rate of athletes returned to their previous sports activities [24]. Patient related factors are difficult to define for successful outcome after ACL-R. These factors are rather complex and influenced by interaction between muscle and knee function, concomitant knee pathologies, effusion, pain, limited range of motion but also by patient’s motivation [4, 34, 36]. Effusion and inferior quadriceps force may reduce the likelihood of return to sport [9]. In contrast, age does not seem to show any impact on clinical outcome however, greater improvement according to KOOS was observed in younger patients after ACL-R [10, 21].

Effusion, muscle function and knee stability are proven as important factors for good clinical outcome following ACL-R, but the impact of these factors following revision ACL-R remain unclear.

It was hypothesized that impaired muscle function, effusion and anteroposterior instability will deteriorate clinical outcome following revision ACL-R.

## Material and methods

The study was approved by the Ethical committee of the University of Jena, Germany (5115–03/17).

Revision ACL-R was performed in 154 patients at the Sports Clinic Erfurt between 2010 and 2015 after failed isolated ACL-R. Four strand STG was used for primary ACL-R in all patients. Revision ACL-R was performed using either STG of the contralateral side or BPTB grafts of the ipsilateral knee. Patients who received single stage isolated revision ACL-R with or without partial meniscus resection were included in the current study. Exclusion criteria were two stage revision surgery, additional surgery except revision ACL-R, concomitant ligament injuries and additional cartilage repair procedures. Seventy knees of seventy patients (13 female and 57 male) out of the 154 patients fulfilled the requirements and were followed retrospectively by an independent examiner who was not involved in patient treatment. The mean follow-up time was 47.8 ± 20.7 months (range, 15 to 84 months) after revision ACL-R. Contralateral STG was used in eleven patients and BTB in 59 patients. Mean age and BMI were 33 ± 9 years and 27 ± 5 kg/m^2^, respectively at the time of follow-up.

Clinical examination was based on the International Knee Documentation Evaluation Form-2000 (IKDC), and Tegner activity scale at the time of follow up (t3) [19, 39]. Tegner activity scale was also recorded prospectively prior to primary (t1) and revision ACL-R (t2).

Cartilage staging according to the Outerbridge classification was recorded during primary and revision ACL-R for all three compartments separately [33].

Instrumented measurement was performed for evaluating the anterior tibial translation using the Rolimeter® (DJO Global, Freiburg) [12]. The circumference of the thigh was measured bilaterally 10 cm and 20 cm proximal to the medial joint line.

### Statistical analysis

Data were given as mean and standard deviation of the mean. Metric scaled numerical variables were analysed using the dependent t-test. For metric scaled data correlations were calculated with Pearson’s correlation coefficient when normal distribution was proven with Shapiro Wilk test. Ordinal scaled data was correlated with Spearman rank correlation coefficient and for dichotomous data with exact Fisher’s test.

Despite significance, correlation for linear models were estimated to be slightly clinically relevant in term of criterion-based validity when it is above 0.4, relevant above 0.5 and strong when higher than 0.6. Factor analysis for estimation of combined loading on a factor seems not feasible in this sample size.

SPSS© Statistics Version 27 (IBM Corporation, Armonk, USA) was used for Mac. *P*-value of < 0.05 was considered statistically significant.

## Results

The Tegner activity scale decreased significantly from 7.8 ± 1.4 points at primary ACL-R (t1) to 7 ± 1.8 points at to revision ACL-R (t2), and to 5.8 ± 1.7 points at the time of follow up (t3) (*p* < 0.001). Forty-nine patients (70%) reported at the time t2 a level of activity comparable to t1. This number dropped to 24 patients (34%) at t3. None of the patients were able to maintain the level of activity when comparing between t1 and t3.

Patient which scored the Tegner activity scale lower at the time of primary ACL-R showed inferior results also at revision ACL-R (t1 to t2, r_s_ = 0.68, *p* < 0.01) but less relevant when considering the assessment between revision ACL-R and latest follow up (t2 to t3, r_s_ = 0.40, *p* < 0.01). Tegner activity scale at the time of follow up (t3) was highly valid for objective deficits, with slightly clinically relevant correlations for effusion (*r* = − 0.47, p < 0.01) and side to side differences in single leg hop test (*r* = − 0.48, *p* < 0.1). The circumference of the operated thighs in comparison to the contralateral one was reduced by 10 ± 9 mm and 17 ± 12 mm at 10 and 20 cm proximal to the medial joint line respectively (Table 1). A significant correlation was found between muscle atrophy and results of a single leg hope test (*r* = 0.32, *p* < 0.03).

**Table 1:** Side to side difference of the thigh at 10 cm and 20 cm above the medial joint space. Reduction in thigh circumference was seen on the operated side

**Side to side difference of thigh circumference**	**10 cm above the media space**	**20 cm above the medial space**
**Side to side difference 10-20 mm**	68.2%	54.5%
**Side to side difference > 20 mm**	3%	25.8%

Minor correlation was found between the Pivotshift test and the Tegner activity scale (*r* = − 0.29, *p* < 0.05). Instrumented AP site-to-site difference of ≥3 mm showed significant impact on the Tegner activity scale (*p* < 0.019).

IKDC was graded as A in three patients (4.3%), B in 35 (50%), C in 29 (41.4%) and D in three (4.3%). Normal or nearly normal knee function was in 54.3% of the patients at the time of follow-up. All parameters of the IKDC are given in detail in Table 2.

**Table 2:** Number of patients and percentage of each parameter according to IKDC

Parameter	Normal (A)	Nearly normal (B)	Abnormal C	Severly abnormal (D)
**Effusion**	46 (65.7%)	20 (28.6%)	4 (5.7%)	0
**Donor site morbidity**	28 (40%)	36 (51.4%)	6 (8.6%)	0
**Crepitus patellofemoral**	22 (31.4%)	0	46 (68.6%)	2 (2.8%)
**Crepitus medial compartment**	56 (80%)	0	13 (18.6%)	1 (1.4%)
**Crepitus lateral compartment**	63 (90%)	0	7 (10%)	0
**Lack of flexion**	37 (52.9%)	31 (44.2%)	2 (2.8%)	0
**Lack of extension**	34 (48.6%)	20 (28.6%)	15 (21.4%)	1 (1.4%)
**Lateral joint opening**	48 (68.6%)	21 (30%)	1 (1.4%)	0
**Medial joint opening**	57 (81.4%)	10 (14.3%)	3 (4.3%)	0
**Lachman test (25° flexion)**	15 (21.5%)	47 (67.2%)	7 (10%)	1 (1.4%)
**Anterior drawer (90° flexion)**	16 (22.9%)	48 (68.6%)	5 (7.1%)	1 (1.4%)
**Ligament evaluation (instrumented side to side difference)**	15 (21.4%)	48 (68.6%)	6 (8.6%)	1 (1.4%)
**Posterior drawer (90°flexion)**	69 (98.6%)	0	0	1 (1.4%)
**Pivot-shift test**	51 (72.9%)	10 (14.3%)	7 (10%)	2 (2.8%)
**External rotation (30° flexion)**	41 (58.6%)	26 (37.1%)	3 (4.3%)	0
**External rotation (90° flexion)**	64 (91.4%)	3 (4.3%)	3 (4.3%)	0
**One leg hop test**	29 (41.4%)	20 (28.6%)	6 (8.6%)	15 (21.4%)

Cartilage lesions were found in 67% of all patients at the time of revision ACL-R in comparison to 38% at the time of primary ACL-R (Table 3). Lesions at the medial femoral condyle of grad II to IV were apparent following primary and revision ACL-R in 19 (27%) and 42 (60%) patients, respectively. The lateral compartment showed grade II to IV lesions in 2 (3%) patients at the time of primary ACL-R and increased to 6 (8,5%) patients at the time of revision. The number of patients with patellofemoral osteoarthritis of grade II to IV increased from 6 (8.6%) to 20 (28%) patients. Patellofemoral osteoarthritis of > grad II was a significant factor for IKDC Grade C and D (*p* = 0.024). Partial medial meniscus resection showed an impact on side-to-side difference in one leg hop test (*p* < 0.001), effusion (*p* < 0.014) and pivot shift (*p* < 0.031), while lateral meniscus resection showed an impact on the pivot shift test only (*p* < 0.017).

**Table 3:** Assessment of cartilage damage during primary and revision ACL-R according to the classification by Outerbridge

Outerbridge-classification	Patellofemoral		Medial tibia		Lateral tibia		Medial femur		Lateral femur	
ACL surgery	primary	revision	primary	revision	primary	revision	primary	revision	primary	revision
**Grade 0**	65	51	71	66	71	69	51	29	69	65
**Grade 1**	0	2	0	0	0	0	1	0	0	0
**Grade 2**	4	6	0	1	0	1	12	18	2	1
**Grade 3**	2	10	0	3	0	1	7	14	0	3
**Grade 4**	0	2	0	1	0	0	0	10	0	2

## Discussion

The most important finding of the study was that joint effusion, AP stability and site-to-site difference in one leg hop test affected significantly the clinical outcome following revision ACL-R. Joint effusion was found in 35% of patients at the time of follow-up. Persistent knee effusion after ACL-R surgery has been reported previously [38]. Effusion significantly reduces quadriceps muscle activation and muscle strength [25, 29]. Thus, it is not surprising that effusion belongs to one of the six important outcome measures for ACL surgery beside giving way, muscle strength, activity and participation and return to sport [26]. According to one-leg-hop-test, 41% of the patients showed nearly normal side to side muscle strength. Even though high percentage of patients presented side-to-side differences in one-leg-hop-test however, no impact on IKDC was found in the current study. Although there was no impact on IKDC, hop-index between the healthy and injured sides are reliable measure for quadriceps muscle function [29]. Significant correlation between quadriceps muscle atrophy and hop-index was found in the current study. The operated side commonly presents an impaired maximal quadriceps muscle strength achieving up to 88% of the none operated, contralateral side [8, 28, 30, 37]. It was shown that higher quadriceps strength asymmetry is more likely in patients with lower quadriceps strength [29]. This might be one of the reasons that only 20% of the patients showed symmetrical knee function 6 months following ACL-R [15]. The difference in thigh circumference may progress after revision ACL-R [41].

Reduction in voluntary quadriceps muscle activation has been reported after primary ACL-R [18, 37]. The reduction of voluntary activation can be improved by ACL-R but remains bilaterally inferior to healthy subjects. Revision ACL-R does not seem to show an additional effect on quadriceps muscle dysfunction [23]. However, quadriceps dysfunction is known as a risk factor for knee osteoarthritis [3, 11].

Knee stability measured by ap translation and Pivotshift-test showed significant impact on clinical and functional outcome after revision ACL-R. Patients with persistent anteroposterior instability or positive Pivotshift-test showed lower scores in the current study based on the Tegner activity scale. None of the patients of the current study received surgery at the periphery of the knee but there is an increasing awareness nowadays for looking at rotation instability prior to revision surgery [20]. Studies have shown that lateral tenodesis or anterolateral ligament reconstruction improve knee stability after revision surgery [7, 13, 35].

Cartilage degeneration is significantly advanced after ACL surgery than in the aged matched healthy population and showed impact on clinical outcome. Increase in cartilage degeneration was observed in all three compartments of the current study. Degeneration in the patellofemoral compartment showed most significant impact on clinical outcome. In contrast, it was reported that the status of articular cartilage makes the most significant impact on successful revision ACL-R after a follow-up time of 5–9 years [32]. Patients’ follow-up of the current study was 4 years, and further progression of OA remains unknown.

Increase in AP instability was found after revision ACL-R. Interestingly, the study by Cristiani et al. did not find any difference in AP stability after primary and revision ACL-R, but lower clinical outcome was observed after revision surgery [8]. Activity of daily life, symptoms and pain according to KOOS showed inferior results following revision ACL-R. On the contrary, increase in side-to-side difference in AP stability was significantly associated with worse outcome according to Tegner activity scale.

The incidence of OA following ACL-R is 12% based on an insurance database after 4 years of follow-up [5]. The odds ratio of developing OA in patient over the age of 35 years was 2.44. There is a significant progression of OA considering the incidence of 43% 10 years after ACL-R [22]. Radiographic knee OA of grade > 2 was detected in 71% of patients at 10 to 15 years of follow-up time underlining the significant increase in the prevalence of OA with time [42]. In contrast, the incidence of OA in healthy subjects is 7.3% in woman and 6.2% in men at a mean age of 41.8 ± 12.9 years and significantly lower than in patients after ACL-R [16]. The incidence of OA after ACL-R is significantly higher than in healthy subjects. Additional damage of both cartilage and subchondral bone can be presumed after revision ACL-R. Other studies also showed worse outcome and a three to four times higher revision rate after revision ACL-R when compared to primary ACL-R [40]. The outcome based on Tegner activity scale was comparable to previous studies [15, 40]. The Tegner activity scale decreased from 8 points prior to ACL-R to 7 points prior to revision ACL-R to 6 points at the time of follow-up considering the minimal detectable change of 1 [17]. Differences between the two groups can be presumed.

In contrast to the MARS group age did not show any impact on clinical and functional outcome [2]. One reason might be the lower median age of 26 yrs. in the cohort of the MARS-study in comparison to the mean age of 33 yrs. in the current study.

The study has certain limitations. First, the study was retrospectively designed. Patients received either STG from the contralateral side or BTB graft from the ipsilateral side. However, according to the MARS-study no difference in clinical outcome was found between STG and BTB graft after revision ACL-R [27]. A meta-analysis of 32 studies showed that no difference exists between hamstring or BTB graft, but STG resulted in better IKDC knee scores [14]. The impact on quadriceps function remains unclear. In the current study, baseline data in terms of muscle function and IKDC were missing.

In conclusion, special attention needs to be paid to joint effusion and knee laxity in patients following revision ACL-R. Degeneration of the patellofemoral compartment might be treated more sustainable during revision surgery with appropriate cartilage procedures. The expectation in relation to patients age needs to be discussed when revision surgery is considered.
